# Mechanism of Thermochromic and Self-Repairing of Waterborne Wood Coatings by Synergistic Action of Waterborne Acrylic Microcapsules and Fluorane Microcapsules

**DOI:** 10.3390/polym14010056

**Published:** 2021-12-24

**Authors:** Xiaoxing Yan, Wenting Zhao, Lin Wang

**Affiliations:** 1 Co-Innovation Center of Efficient Processing and Utilization of Forest Resources, Nanjing Forestry University, Nanjing 210037, China; 2 College of Furnishings and Industrial Design, Nanjing Forestry University, Nanjing 210037, China; zhaowenting@njfu.edu.cn (W.Z.); wanglin@njfu.edu.cn (L.W.)

**Keywords:** fluorane microcapsule, waterborne acrylic resin microcapsules, coating film, synergistic action

## Abstract

The fluorane thermochromic microcapsules and waterborne acrylic resin microcapsules were added into waterborne coatings at the same time to prepare intelligent waterborne coating film with dual functions of color change and self-repairing. The coating film prepared by adding 15.0% fluorane microcapsules and 5.0% waterborne acrylic resin microcapsules to the primer at the same time had better comprehensive properties. At this time, the coating film changed from yellow to colorless. The repair rate of the coating film was 58.4%. When the temperature was lower than 32 °C, waterborne acrylic resin microcapsules can improve the thermochromic performance of the coating film with fluorane microcapsules. Waterborne acrylic resin microcapsules can alleviate the color change of coating film with fluorane microcapsules. The fluorane microcapsules can improve the self-repairing performance of coating film with waterborne acrylic resin microcapsules. The results lay a theoretical and technical foundation for multifunctional coating film.

## 1. Introduction

Due to the unique characteristics of wood, such as wet expansion, dry shrinkage, porosity, and anisotropy, the actual use of wood will be affected [1,2,3]. Coatings on the surface of wood can make up for these defects and play a role in protection and beautification [4]. In addition, wood coatings can not only stabilize the performance of wood materials, but also have the functions of anti-corrosion, flame-retardant, and fragrance retention [5]. Among many kinds of wood coatings, waterborne coatings are very popular in wood finishing because of their advantages of protecting the environment, and being pollution-resistant and fast-drying [6]. However, the comprehensive properties of waterborne coatings are inferior to solvent-based coatings, such as insufficient film fullness, low hardness, high price, poor water resistance, and easy occurrence of microcracks on furniture surfaces.

Microcapsules with repairing agent as core material are called self-repairing microcapsules. Self-repairing microcapsules can intelligently monitor the external environment, and when the matrix material is damaged, microcapsules can automatically repair it. Du et al. [7] studied the self-repairing ability of microencapsulated mortar containing toluene diisocyanate with different shell compositions at 10 °C, 30 °C, 50 °C, and 60 °C. The self-repairing ability of mortar containing different microcapsules increased with the increase of temperature from 10 °C to 50 °C. When the ambient temperature reaches 50 °C, the surface cracks of three kinds of microcapsule mortars can completely heal themselves within 5 h. Wu et al. [8] prepared microcapsules with triethylene glycol dimethacrylate (TEGDMA) and n,n,N-dihydroxyethylp-toluidine (DHEPT) as the core materials of therapeutic fluid and polyurea formaldehyde (PUF) as the wall material. Microcapsules were added to the composite materials, and dental composites with three advantages: self-repair, antibacterial, and remineralization after fractures were developed. Ataei et al. [9] encapsulated green acrylic epoxy resin and its sulfhydryl curing agent in the PMMA shell by electrospray technology, and prepared binary components of the self-repairing epoxy system. The results showed that when the concentration of the binary capsule was 1.0%, the self-healing efficiency was the highest (78.0%), and can be used as a new functional unit in the wood field, bringing new favorable conditions for the growth of wood functional materials. However, these studies have introduced new components, which were inconvenient to analyze the mechanism and complicated to operate. Moreover, the self-repairing properties of microcapsules were mostly characterized on steel, and the research on wood substrate was not sufficient.

Thermochromic coatings are a kind of intelligent coating which can measure and express the surface temperature and temperature distribution of objects through color change. Thermochromic microcapsules were widely used in machinery, camouflage, anti-counterfeiting, and other fields. The thermochromic microcapsule is a kind of material with intelligent response, which can change its color with the change of ambient temperature [10]. That is, when raised to a specific temperature, the microcapsules can change from one color to another, and then change to the original color after the temperature drops [11]. Cao et al. [12] assembled inorganic pigments and two kinds of thermochromic microcapsules with different response temperatures onto a filter paper by extraction and filtration, and then sealed the whole structure with epoxy resin to achieve reversible color changes. Ma et al. [13] designed a new type of thermochromic microcapsules by combining thermochromic materials with optimized dispersive-cationic dyes. With the change of environmental temperature, the color of phase change materials can change reversibly between different tones, showing a more beautiful color than ordinary thermochromic materials. Wu et al. [14] prepared thermochromic hydrogels by encapsulated three components of temperature-responsive microcapsules containing a chromogenic agent, cryptochromic agent, and solvent. It had the advantages of bright color, fast response, and long service life. A soft display and camouflage technology using thermochromic hydrogels as the discoloring phase and an electric heating wire as a driving phase was proposed. However, these studies were limited to discoloration performance, and other performances were not deeply explored and optimized on the basis of discoloration.

Fluorane thermochromic microcapsules have the advantages of low discoloration temperature, high sensitivity, and environmental friendliness. The core components of waterborne acrylic resin microcapsules are the same as waterborne coatings, which are safe and nontoxic, and can be cured at room temperature. More importantly, it is easy to analyze without introducing foreign components. In the early stage, we studied the influence of waterborne acrylic resin microcapsules on the performance of waterborne film [15], and also studied the influence of thermochromic microcapsules on the performance of waterborne coating film [16]. However, the influence of the mixed addition of fluoran microcapsules and waterborne microcapsules on the coating film has not been explored.

In this paper, the thermochromic mechanism of fluorane microcapsules assisted by waterborne acrylic resin microcapsules and the self-repairing mechanism of waterborne acrylic resin microcapsules assisted by fluorane microcapsules were discussed, and the synergistic action of waterborne acrylic microcapsules and fluorane microcapsules was explored. It provides a new idea for the development of multifunctional waterborne coating film.

## 2. Materials and Methods

### 2.1. Materials

The 38.0% formaldehyde solution (M_w_: 30.03 g/mol, CAS No.: 50-00-0), urea (M_w_: 60.06 g/mol, CAS No.: 57-13-6), triethanolamine (M_w_: 149.18 g/mo, CAS No.: 102-71-6), sodium dodecylbenzene sulfonate (M_w_: 348.48 g/mol, CAS No.: 25155-30-0), citric acid monohydrate (M_w_: 210.14 g/mol, CAS No.: 5949-29-1), and N-octanol (M_w_: 130.228 g/mol, CAS No.: 111-87-5) were provided by Guangzhou Jiangshun Chemical Technology Co., Ltd., Guangzhou, China. Basswood (95 mm × 40 mm × 5 mm) was provided by Yunhe Youlin Wood Industry Co., Ltd., Lishui, China. Basswood was pre-sanded, with a smooth surface and uniform color. Thermochromic microcapsules (fluorane microcapsules) were provided by Shenzhen Dongfang Color Technology Co., Ltd., Shenzhen, China. The discoloration temperature was 31 °C. The main components were polycyanamide formaldehyde resin, 1,2-benzo-6-diethylaminofluorane, methyl palmitate, and ethyl stearate. Dulux waterborne primer coatings and Dulux waterborne topcoat coatings were provided by Changsha Haodu Coatings Co., Ltd., Changsha, China, which mainly consist of waterborne acrylic emulsion, polyurethane emulsion, additives, and water, with a solid content of about 30.0%.

### 2.2. Preparation of Microcapsules

A total of 20.0 g of urea and 27.0 g of 38.0% formaldehyde were added into a beaker according to the molar ratio of 1:1, placed in a magnetic stirrer to be completely dissolved by stirring, and triethanolamine was slowly added to adjust the pH to about 8.0. A slightly viscous transparent urea formaldehyde prepolymer solution was obtained by stirring at 1200 rpm for 2.0 h in a water bath at 75 °C. The 135.6 mL of distilled water and 1.3 g of white powder of sodium dodecyl benzene sulfonate were stirred until completely dissolved, which was used as an emulsifier. The 17.5 g of waterborne acrylic resin coatings was added to the emulsifier, stirred at 1200 rpm in a 65 °C constant temperature water bath for 25 min, and we then added 1–2 drops of n-octanol to defoam. The magnetic stirrer speed was adjusted to 400 rpm, urea-formaldehyde prepolymer was dropped into the core emulsion, citric acid crystals were gradually added, and the pH was adjusted to 2.5. It was reacted at 70 °C for 3 h. After that, it was filtered by suction and washed with distilled water and absolute ethyl alcohol to remove excess emulsifier and uncoated core and wall materials, then dried at 80 °C for 4 h to obtain a white microcapsule powder.

### 2.3. Preparation of Thermochromic Self-Repairing Dual-Function Coating Film

Three-factor and two-level orthogonal experiments were designed with “content of fluorane microcapsules”, “content of waterborne acrylic resin microcapsules”, and “adding method of microcapsules”, as shown in Table 1. Excessive powder addition will affect the roughness and gloss of the coating film, which was not conducive to industrial applications. Therefore, the content of color-changing microcapsules was set at 10.0–20.0%, and the content of self-repairing microcapsules was set at 5.0–15.0% [17]. The ingredients of the waterborne coatings with two types of microcapsules were shown in Table 2. The samples 1–4# were the materials corresponding to the orthogonal experiment. The samples 6–10# were an optimized experiment based on an orthogonal experiment. The samples 5#, 11# and 12# were blank control samples. The microcapsules and waterborne coatings with corresponding quality were weighed, stirred evenly, coated on the surface of the Basswood, dried at room temperature until the quality of the coating film no longer changed, and the thickness of the prepared dry film was about 60.0 µm.

### 2.4. Testing and Characterization

Coating film samples were heated by a YOUYUE946C constant temperature heating plate (Shenzhen Youyue Jinggong Trading Co., Ltd., Shenzhen, China). At the same time, a 2003Y thermometer (Zhongshan Leyi Electronic Technology Co., Ltd., Zhongshan, China) was used to measure the temperature change of the coating film surface. The SEGT-J portable colorimeter (Kunshan Lugong Precision Instrument Co., Ltd., Suzhou, China) was used to measure the color difference change of the coating film at 16–40 °C. *L* represents the lightness, *a* represents the red-green value, and *b* represents the yellow-blue value. The larger the L value, the brighter the color; the larger the a value, the redder the color; the larger the b value, the yellower the color. The change of chromaticity parameters at different temperatures during heating was recorded from 16 °C. According to Hunter’s color difference equation, the variation trend of color difference of samples at different temperatures was calculated [18].

(1) $$\begin{array}{cc} color & difference \end{array}=\sqrt{{\left(L_{1}-L_{2}\right)}^{2}+{\left(a_{1}-a_{2}\right)}^{2}+{\left(b_{1}-b_{2}\right)}^{2}}$$

The hardness of the coating film was measured by an AS-120P portable pencil hardness tester (Qingdao Lintuo Environmental Protection Technology Co., Ltd., Qingdao, China) and 6H-6B pencil. When testing the hardness, the angle between the pencil and the coating film was 45°, the pencil was scratched under a load of 1.0 kg, and the pencil hardness without scratching the coating film indicated the hardness of the coating film [19]. The QFH hundred-grid knife coating cross-cutting device (Shanghai Liangyan Intelligent Technology Co., Ltd., Shanghai, China) was selected to test the coating adhesion [20]. According to GB/T 1732-1993 [21], the QCJ-50 film impact tester (Changzhou Jonah Electromechanical Technology Co., Ltd., Changzhou, China) was selected to measure the coating film impact resistance. The weight was fixed at the required height with the control screw, and its height was read out through the positioning mark. The controller screw was pressed down, so that the connected weight falls down and acts on the sample. Then the hammer body was lifted, the tested sample was taken out, and the coating on the surface of the sample was observed with a magnifying glass. The impact strength of the coating film was expressed by the maximum height at which a heavy hammer impacts the coating film without causing damage. The elongation at break of the coating film was measured by an AG-IC100KN precision electronic universal testing machine. The waterborne coatings were coated on the glass substrate. After the coating film was completely dried, it was placed in clear water to be peelable. After the coating film was peeled off, it was dried at room temperature. During the test, the two ends of the coating film were clamped with clamps to ensure that the coating film will not slide. The two ends of the coating film were clamped by clamps, so that the coating film was completely straightened but not stressed. The vernier caliper was used to record the distance between clamps, which is *L_0_*. The machine was started and stopped when the coating film broke. The distance between clamps after the coating film broke was measured, which is *L_1_*. The elongation at break (*P*) of the coating film was calculated according to the following formula:

(2) $$P=\frac{L_{1}-L_{0}}{L_{0}}\times 100\%$$

According to GB/T 4893.1-2005 [22], 15.0% NaCl solution, 70.0% medical ethanol, detergent, and red ink were selected to measure the liquid resistance of the coating. The filter paper was soaked in the test solution for 30.0 s, then taken out with tweezers and placed in the experimental area. Tempered glass cover was used to cover the test area. The filter paper was taken off after 24 h. Absorbent paper was used to absorb the residual liquid on the surface of the coating film. After 30 min, the damage of the experimental area of the sample was checked to determine the liquid resistance grade of the coating film. The 202-00 oven (Taizhou Junqian Electric Heating Equipment Co., Ltd., Taizhou, China) and JY-UV-1300 ultraviolet weathering test box (Dongguan Jingyu Environmental Testing Equipment Co., Ltd., Dongguan, China) were used to carry out the aging resistance experiment of coating film. The oven temperature was set at 120 °C and 160 °C, the coating film was put into the oven, and the color difference of the coating film was tested every 8 h for 40 h. The color difference of the coating film in the ultraviolet weathering test box was tested every 40 h for 200 h in total. The self-repairing rate was proved by a scratch experiment. After the coating film was scratched by a utility knife, the crack width of the coating film was observed by a microscope, and it was allowed to stand at room temperature for 7 days. Then the crack width of the coating film was observed again, and the repairing rate of the coating film was calculated according to the formula (3). *A_original_* indicates the crack width of the coating film before repair, and *A_healed_* indicates the crack width of the coating film after self-repairing.

(3) $$\sigma =\frac{A_{original}-A_{healed}}{A_{original}}\times 100\%$$

The microstructure of the coating film was observed by an Axio Scope A1 optical microscope and Quanta 200 scanning electron microscope (FEI Company, Hillsboro, OR, USA). Chemical composition of the coating film was analyzed by a VERTEX 80v Fourier infrared spectrometer (Germany BRUKER Co., Ltd., Karlsruhe, Germany). All experiments were repeated four times, and the error was less than 5.0%.

## 3. Results and Discussion

### 3.1. SEM and FTIR of the Microcapsules

**Figure 1:**
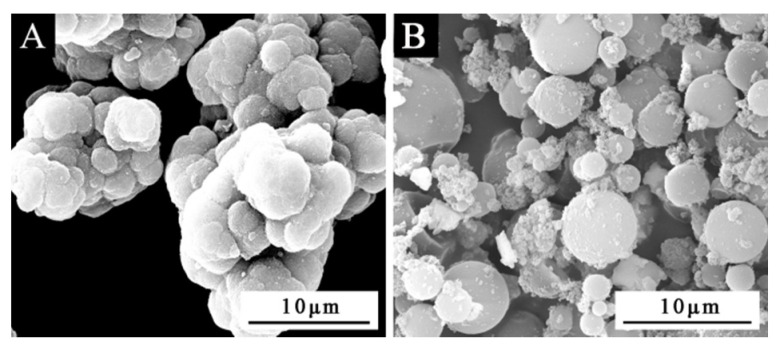
SEM diagram of microcapsules: (**A**) waterborne acrylic resin microcapsules, (**B**) fluorane microcapsules.

**Figure 2:**
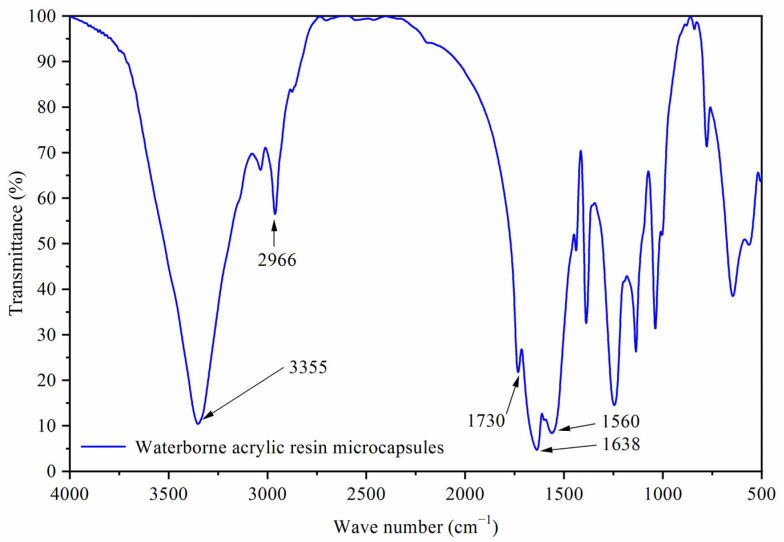
Infrared spectrum of waterborne acrylic resin microcapsules.

**Figure 3:**
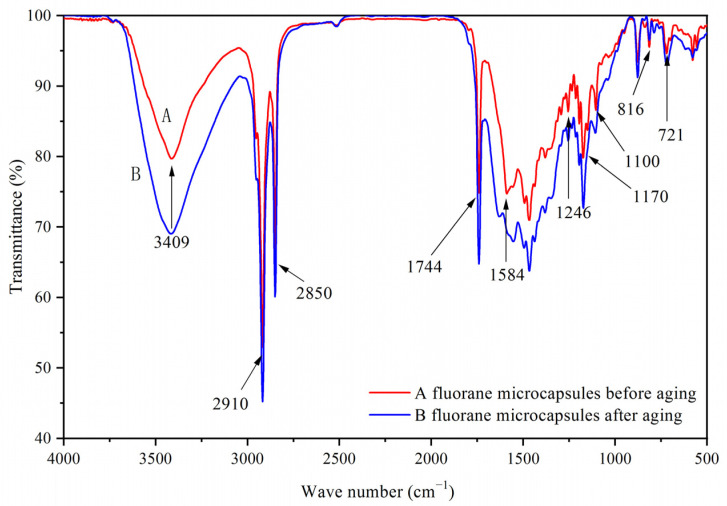
Infrared spectrum of fluorane microcapsules.

Figure 1A was a scanning electron microscope diagram of waterborne acrylic resin microcapsules, and Figure 1B was a scanning electron microscope diagram of fluorane microcapsules. It can be seen that the microcapsules were roughly spherical with a particle size of about 4.0 µm. The agglomeration of the waterborne acrylic resin microcapsules was relatively serious, and the fluorane microcapsules were more uniformly dispersed. Figure 2 was the infrared spectrum of waterborne acrylic resin microcapsules. The special absorption peaks around 3355 cm^−1^, 1560 cm^−1^ were the absorption peaks of N–H, C–N in urea-formaldehyde resin [23,24]. The 1730 cm^−1^ represented the characteristic peak of C=O in waterborne acrylic resin [25]. Therefore, it was proved that waterborne acrylic resin microcapsules had been successfully prepared. The FTIR of fluorane microcapsules was shown in Figure 3. The fluctuation at 3409 cm^−1^ was caused by –NH and –OH stretching vibration [26], and the stretching vibration absorption peak and bending vibration absorption of triazine ring were located at 1584 cm^−1^ and 816 cm^−1^, respectively [27]. The expansion and contraction vibration absorption of C–O–C appeared at 170 cm^−1^, 1100 cm^−1^ and 1246 cm^−1^ [28]. The stretching vibration of carbonyl group in conjugated chromogenic structure appeared at 1744 cm^−1^, which indicated that 1,2-benzo-6-diethylamino fluorane in fluorane microcapsule core material existed in conjugated chromogenic structure at low temperature [29]. The extensional vibration of –CH_3_ and –CH_2_ occured at 2910 cm^−1^ and 2850 cm^−1^ [30]. In-plane bending vibration of –CH_2_ was at 721 cm^−1^ [31]. Infrared spectrum proved that the wall material of fluorane microcapsule was melamine resin, and the core material was a color-changing compound.

### 3.2. Orthogonal Experimental Analysis

**Figure 4:**
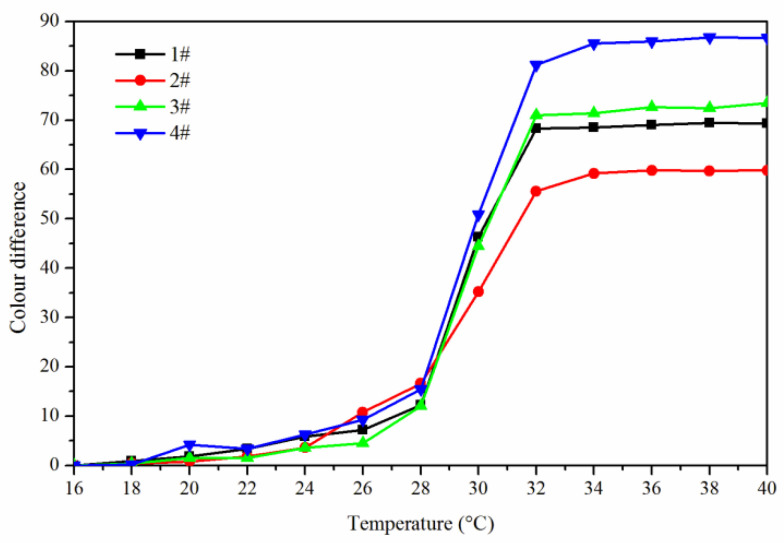
The effect of temperature (16 °C to 40 °C) on the color difference of the coating film.

The effect of temperature (16 °C to 40 °C) on the color difference of the coating film with the addition of fluorane microcapsules was shown in Figure 4. It can be seen from Figure 4 that within the range of 16–30 °C, the increasing amplitude of color difference of coating film had little tendency. When the temperature rose from 30 °C to 32 °C, the increasing amplitude of color differences of coating film became larger and tended to a maximum at 32 °C, indicating that the orthogonal 1–4# thermochromic temperature range was 30–32 °C, and thermoreversible discoloration occurred at 32 °C.

In order to make the coating film have the best thermochromic performance, the influence of these factors on its color difference performance was mainly explored, so the results of 16–32 °C color difference were brought into the orthogonal analysis table for analysis, as shown in Table 3. Orthogonal experiment and range results showed that “fluorane microcapsule content” was the primary factor affecting the color difference of coating film. According to mean value 1 and mean value 2, it can be further known that “waterborne acrylic resin microcapsule content was 5.0%, and fluorane microcapsules and waterborne acrylic resin microcapsules were added to primer at the same time” had greater influence on the color difference of coating film. Therefore, when the orthogonal experiment was optimized in the next step, the content of waterborne acrylic resin microcapsules was fixed at 5.0%, the fluorane microcapsules and waterborne acrylic resin microcapsules were added to the primer at the same time, and the content of fluorane microcapsules (0%, 5.0%, 10.0%, 15.0%, 20.0%, 25.0%, 30.0%) was studied.

### 3.3. Single-Factor Experiment Optimization of Fluorane Microcapsule Content

**Figure 5:**
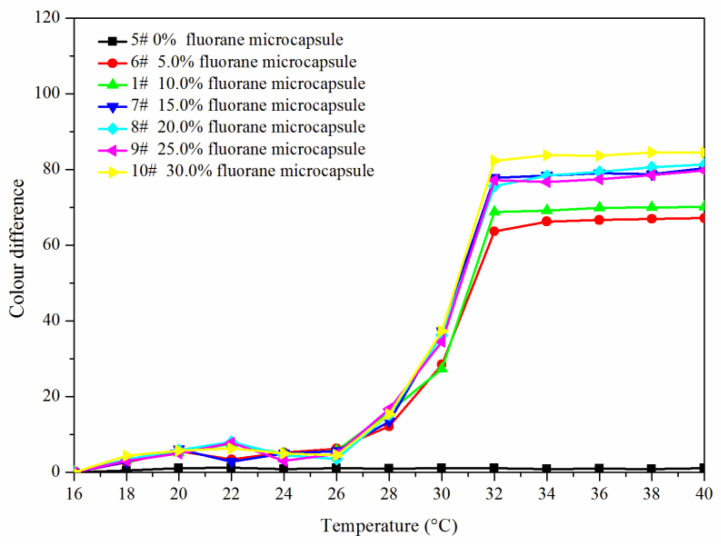
Rising temperature on the color difference of the coating film.

**Figure 6:**
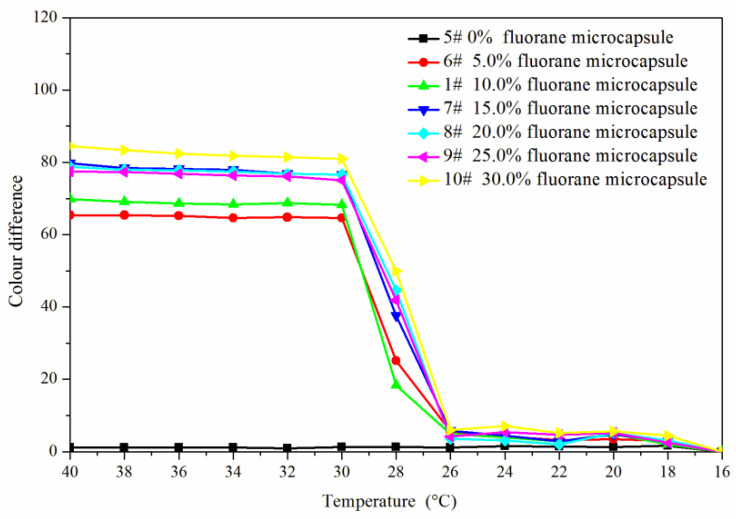
Falling temperature on the color difference of the coating film.

Figure 5 and Figure 6 showed the effect of rising and falling temperature on the color difference of the coating film. The color difference of the coating film without fluorane microcapsules did not change with temperature, so it had no thermochromic effect. The color difference of the coating film with fluorane microcapsules changed with temperature, which had thermochromic effect. When the temperature was 16–26 °C, the color difference of the coating film was all below 10.0, and there was no obvious color difference. When the temperature was higher than 28 °C, the color difference of the coating film increased obviously, and when the temperature was higher than 32 °C, the color difference was basically unchanged. It showed that the coating film completely changed color at 32 °C. When the content of fluorane microcapsules was 15.0–30.0%, the color difference was obvious and the thermochromic effect was better.

The influence of fluorane microcapsule content on the mechanical properties (elongation at break, impact resistance, adhesion, and hardness) of the coating film was shown in Table 4. With the increase of fluorane microcapsule content, the impact resistance of the coating film also increased. This was because compared with the coating film without microcapsules, when the coating film with microcapsules was impacted, the microcapsules can play a buffering role, which can transfer the impact force to the surrounding of the wall material and reduce the internal stress of the matrix material [32]. When the content of fluorane microcapsule increased from 0% to 30.0%, the adhesion grade of the coating film was 0, and the adhesion performance was excellent, which indicated that the increase of fluorane microcapsule content in primer will not change the excellent adhesion of the original coating film. The elongation at the break of the coating film was arched, and the best elongation at the break of the coating film was 37.9% when the amount of fluorane microcapsule was 5.0%. This was because the wall materials of the two kinds of microcapsules in the primer were urea formaldehyde resin and melamine formaldehyde resin, which played a toughening role in the tensile process. More importantly, the released of the waterborne acrylic resin in the core material under external force can alleviate the generation of cracks, so the toughness of the coating film will be increased, and the elongation at break will be increased. However, with the increase of the content, the microcapsules formed agglomeration in the coating film, which reduced the flexibility of the coating film.

**Figure 7:**
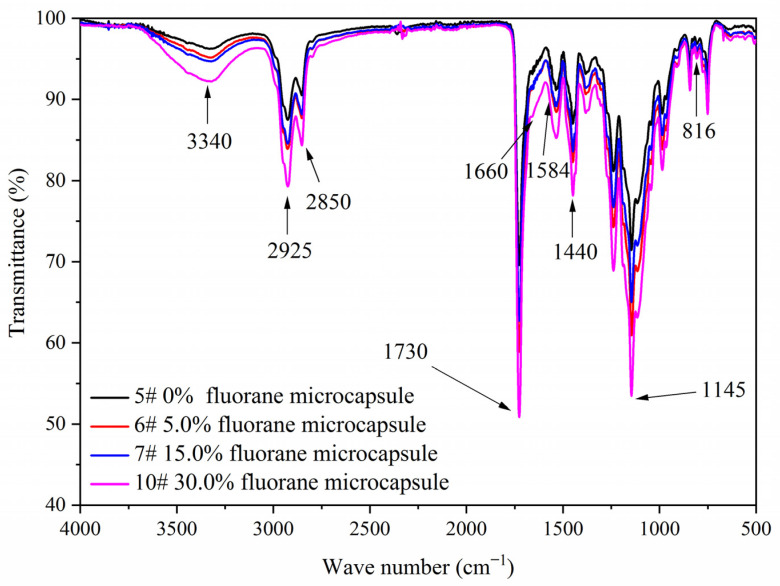
FTIR of the coating film with different fluorane microcapsule content.

Figure 7 shows the FTIR of the coating film with different contents of fluorane microcapsules and 5.0% waterborne acrylic resin microcapsules. Tensile vibration of –NH and –OH was located at 3340 cm^−1^, tensile vibration of –CH_3_ was located at 2925 cm^−1^, and the stretching vibration absorption peak and bending vibration absorption of triazine ring were located at 1584 cm^−1^ and 816 cm^−1^, respectively, and the stretching vibration of C=O in urea-formaldehyde resin was located at 1660 cm^−1^. The absorption of carbonyl stretching vibration was located at 1730 cm^−1^, which not only represented the characteristic peak of C=O in the core material of waterborne acrylic resin microcapsules, but also represented the characteristic peak of C=O in the core material of fluorane microcapsules (1,2-benzo-6-diethylamino fluorane). No peak disappeared or appeared, indicating that there was no chemical change between microcapsule and waterborne coatings.

### 3.4. Analysis of the Discoloration Mechanism of Fluorane Microcapsules by Synergistic Effect of Waterborne Acrylic Microcapsules

**Figure 8:**
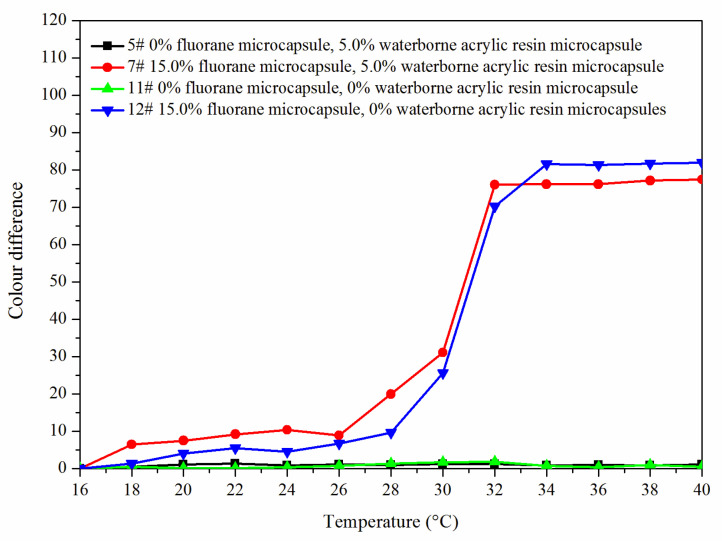
The color difference of the coating with the increased temperature.

**Figure 9:**
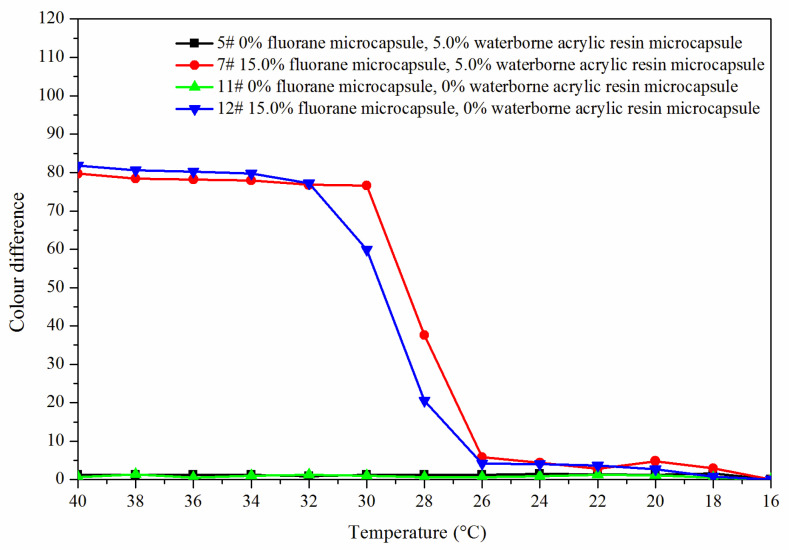
The color difference of the coating with the decreased temperature.

Figure 8 shows the color difference trend of the best sample 7# and blank samples 5#, 11# and 12# at elevated temperature. The color difference of coating film (5# and 11#) without fluorane microcapsules did not change with temperature and had no thermochromic effect. The color difference of the coating film with fluorane microcapsules (7# and 12#) reached the maximum at 32 °C, indicating that the coating film was completely discolored. Combined with Figure 9, it can be concluded that the coating film added with fluorane microcapsules had a reversible thermochromic phenomenon.

**Figure 10:**
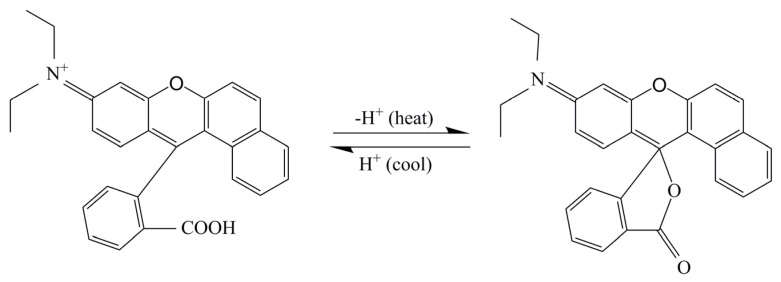
Thermochromic mechanism of fluorane thermochromic microcapsules.

Figure 10 shows the thermochromic mechanism of fluorane thermochromic microcapsules. The central carbon atom of 1,2-benzo-6-diethylaminofluoran had a certain electrophilicity. In the presence of a solvent, the developer dissociated from H+ after accepting electrons, and then attacked the central carbon atom of fluoran. The lactone ring was broken, and the central carbon atom changed from the SP3 hybrid state to the SP2 hybrid planar configuration, forming a large conjugated structure, which caused the emission and absorption spectra to change, forming a quinone structure, thus showing yellow. When the temperature was higher than the color change temperature, the central carbon atom of 1,2-benzo-6-diethylamino fluorane formed a ring to form a SP3 hybrid tetrahedral structure. The molecules were not in the same plane and could not form a common system, so they were colorless [33]. The addition of fluorane microcapsules in the waterborne primer or topcoat did not affect its thermochromic properties.

**Figure 11:**
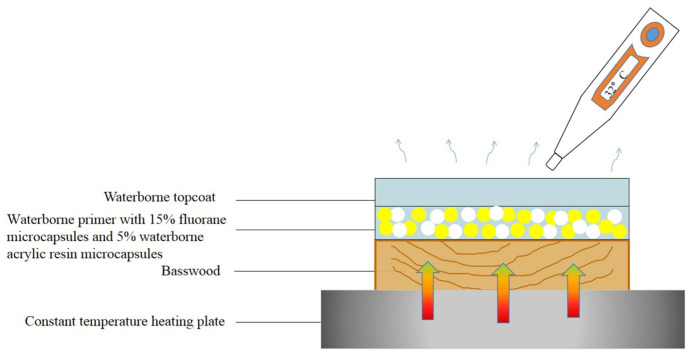
Schematic diagram of the thermochromic mechanism of fluorane with the synergistic effect of waterborne acrylic microcapsules.

Figure 11 is a schematic diagram of the thermochromic mechanism of fluorane with a synergistic effect of waterborne acrylic microcapsules. It can be seen from Figure 8 and Figure 9 that when the temperature was lower than 32 °C, the color difference of the coating film of 7# with 15.0% fluorane microcapsules and 5.0% waterborne acrylic resin microcapsules was greater than that of 12# with only 15.0% fluorane microcapsules. This was because when the sample was heated on the heating plate, the temperature was transferred from the bottom of Basswood to the surface of the coating film, and the heat will be gradually lost, so the temperature below the coating film was higher than that on the surface of the coating film. Waterborne acrylic resin microcapsules form a barrier layer, which was not conducive to heat dissipation and has a stronger heat preservation effect, so that fluorane thermochromic microcapsules can reach the discoloration temperature ahead of time, so waterborne acrylic microcapsules accelerate the discoloration effect to some extent (samples 7# and 12# near 32 °C in Figure 8). When the temperature was higher than 32 °C, the white waterborne acrylic resin microcapsules weaken the transparency of the coating film, so the chromaticity value of 7# coating film was weakened to some extent. Therefore, when the temperature was lower than 32 °C, the waterborne acrylic resin microcapsules could be used to synergize the thermal discoloration of the coating film with fluorane microcapsules.

### 3.5. Aging Resistance Test of Coating Film

**Figure 12:**
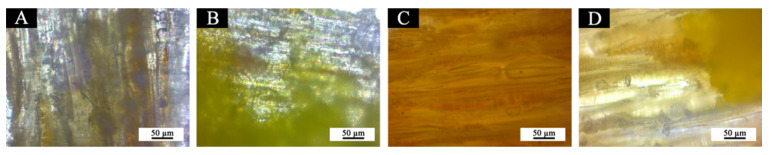
Optical microstructure diagram of (**A**) 5#, (**B**) 7#, (**C**) 11#, and (**D**) 12#.

In order to explore the relationship between fluorane microcapsules, waterborne acrylic resin microcapsules, waterborne coatings and Basswood, the blank samples 5# (0% fluorane microcapsules, 5.0% waterborne acrylic resin microcapsules), 11# (0% fluorane microcapsules, 0% waterborne acrylic resin microcapsules), 12# (15.0% fluorane microcapsules, 0% waterborne acrylic resin microcapsules), and the best sample 7# (15.0% fluorane microcapsules, 5.0% waterborne acrylic resin microcapsules) were sliced and placed under an optical microscope for observation and analysis. Figure 12A–D was an optical microstructure diagram of 5#, 7#, 11#, and 12#, in turn. The arrangement of the holes in the duct wall of Basswood seen in the four figures was parallel. It can be seen from Figure 12B,D that the waterborne coating film of fluorane microcapsules on the surface of Basswood was well-dispersed, which may be due to its small particle size. It can be seen from Figure 12A,B that the waterborne acrylic resin microcapsule particles in the primer were larger, which may be caused by some agglomeration in the coating film.

**Figure 13:**
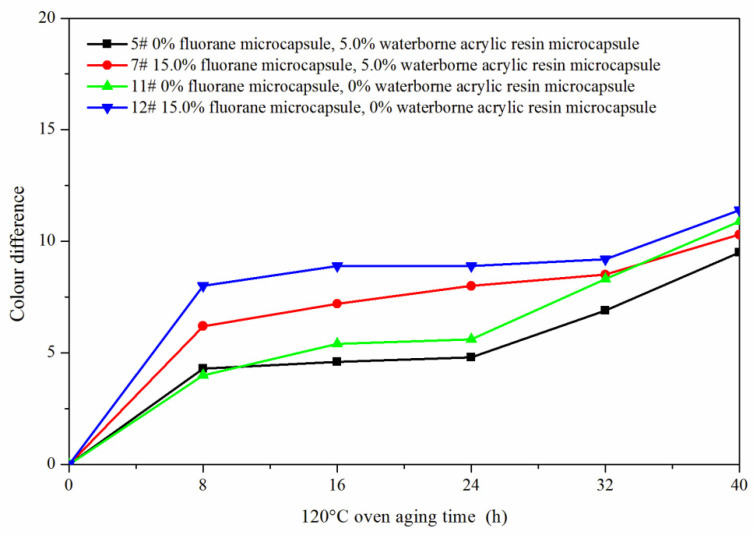
Effect of aging time on color difference of film at 120 °C in oven.

**Figure 14:**
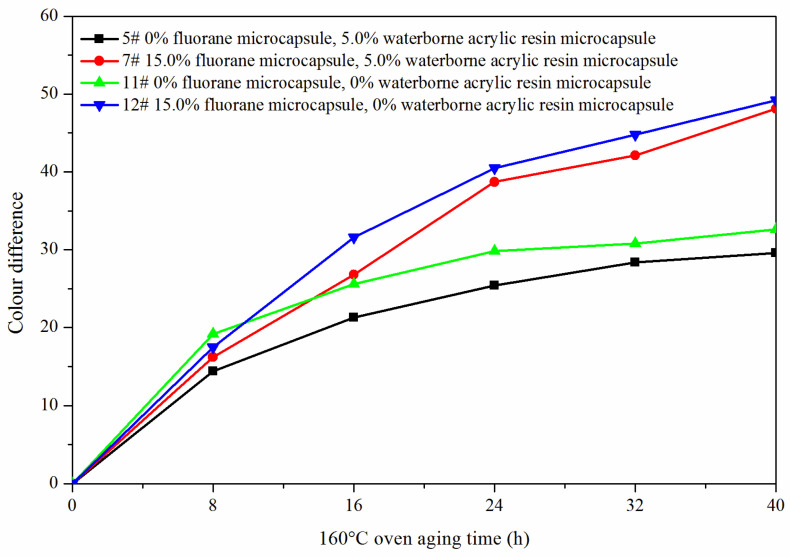
Effect of aging time on color difference of film at 160 °C in oven.

**Figure 15:**
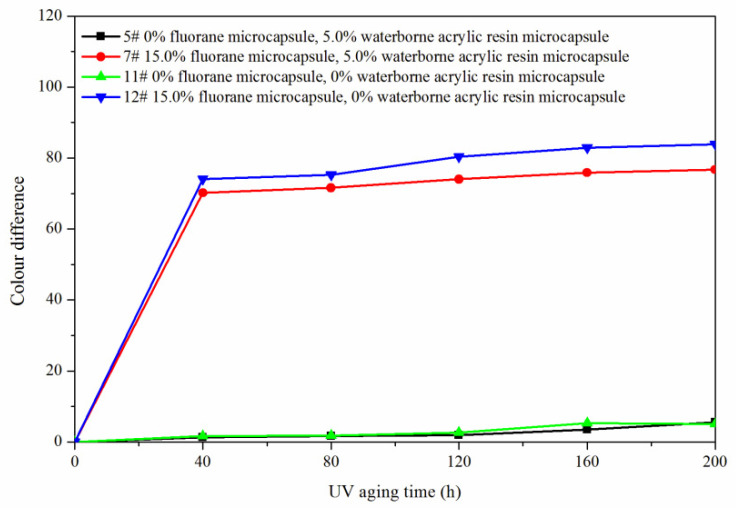
Effect of UV aging time on the color difference of film.

The samples 5#, 11#, 12#, and 7# were tested for aging resistance in three environments: 120 °C, 160 °C, and ultraviolet weathering and the surface conditions of coating film were observed. After aging at 120 °C (Figure 13), the color difference of 5# increased to 9.5 with the increase of heating time. With the increase of heating time, the color difference of 7# increased to 10.3, the color difference of 11# increased to 10.9 and the color difference of 12# increased to 11.4. After aging at 160 °C (Figure 14), the color difference of 5# increased to 29.6 with the increase of heating time. With the increase of heating time, the color difference of 7# increased to 48.1, the color difference of 11# increased to 32.6 and the color difference of 12# increased to 49.2. After aging in the ultraviolet weatherproof test chamber (Figure 15), the color difference of 5# increased to 5.6 with the increase of heating time. With the increase of heating time, the color difference of 7# increased to 76.7, the color difference of 11# increased to 5.1 and the color difference of 12# increased to 83.8.

The results showed that the color difference of the same coating film increased with the aging time. After aging, the color difference of samples only added with fluorane microcapsules was larger, which indicated that fluorane microcapsules were unstable during aging and cannot keep the chromaticity value of samples. However, the aging color difference of the coating film added with both waterborne acrylic resin microcapsule and fluorane microcapsule tended to slow down. This was because the coating film may have microcracks in the aging process, and the core (repairing agent) of the waterborne acrylic resin microcapsule flowed out, which can repair the microcracks and inhibit the surface damage. On the other hand, the increase of color difference may be due to the color change of wood in the aging environment, which made the color difference of coating film bigger.

**Figure 16:**
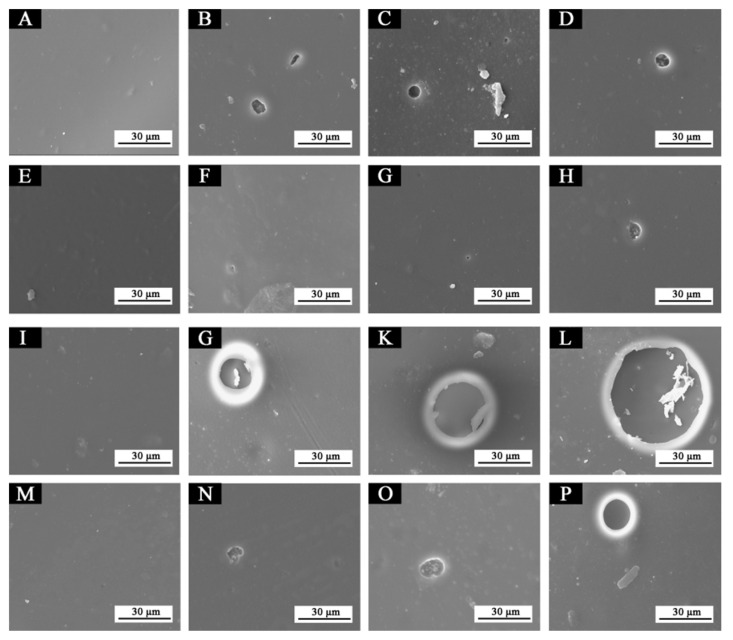
SEM of paint films before and after aging in different environments: (**A**) 5#, (**B**) 5#-120 °C, (**C**) 5#-160 °C, (**D**) 5#-UV, (**E**) 7#, (**F**) 7#-120 °C, (**G**) 7#-160 °C, (**H**) 7#-UV, (**I**) 11#, (**J**) 11#-120 °C, (**K**) 11#-160 °C, (**L**) 11#-UV, (**M**) 12#, (**N**) 12#-120 °C, (**O**) 12#-160 °C, (**P**) 12#-UV.

Figure 16 showed SEM images of samples 5#, 7#, 11#, and 12# before and after aging in different aging environments. After aging at 120 °C, tiny bubbles began to appear in the 5# coating film. After aging at 160 °C and in the ultraviolet weathering test chamber, the number and diameter of bubbles increased slightly, but it was not obvious. No obvious bubbles or cracks appeared on the surface of the 7# coating after aging at 120 °C and 160 °C, and the coating film was in good condition. Micro-bubbles appeared, but were not obvious after aging in the ultraviolet weathering test chamber. After aging at 120 °C, in the 11# coating film larger bubbles began to appear, and after aging at 160 °C and in the ultraviolet weathering test chamber, the bubble diameter increased sharply, which indicated that the coating film was obviously damaged. After aging at 120 °C, tiny bubbles began to appear in the coating film 12#, and the bubble diameter increased slightly at 160 °C. After aging in the ultraviolet weathering test chamber, the bubble diameter increased. The results showed that the waterborne coating film with fluorane microcapsules or waterborne acrylic resin microcapsules had a certain crack inhibition effect, and the coating film with waterborne acrylic resin microcapsules had a better crack inhibition effect. This may be because no matter what type of microcapsules, their shape was similar to a spherical shape, which can be better and more evenly distributed in the coating film, so as to be closely combined with the coating film to form a relatively dense protective coating film. Therefore, it can block part of the damage of high temperature and ultraviolet when aging in a more timely manner, and can better adapt to the invasion of environmental factors. On the other hand, the coating film added with waterborne acrylic resin microcapsules may break during aging and the core material repair agent (waterborne acrylic resin) released, thus inhibiting the large-area damage of the coating film microstructure [34,35].

**Figure 17:**
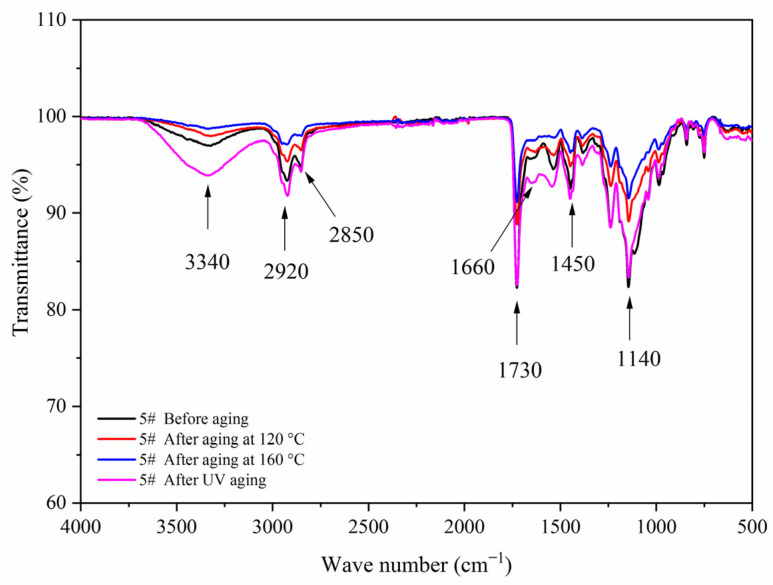
FTIR of sample 5# before and after aging.

**Figure 18:**
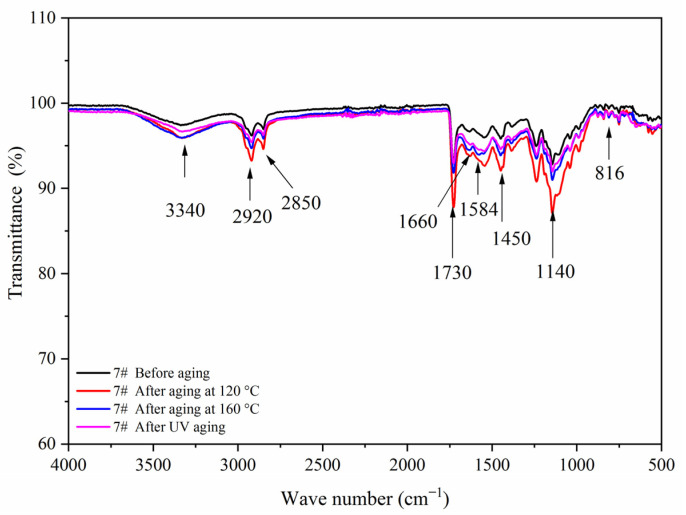
FTIR of sample 7# before and after aging.

**Figure 19:**
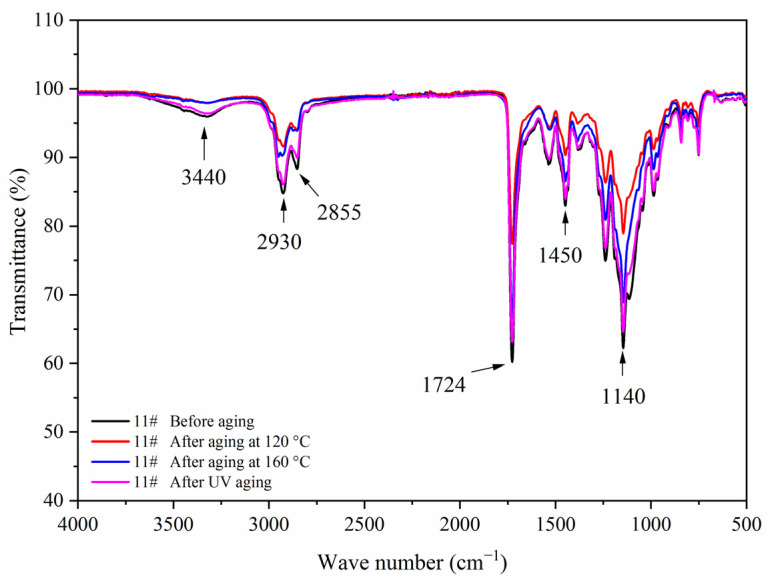
FTIR of sample 11# before and after aging.

**Figure 20:**
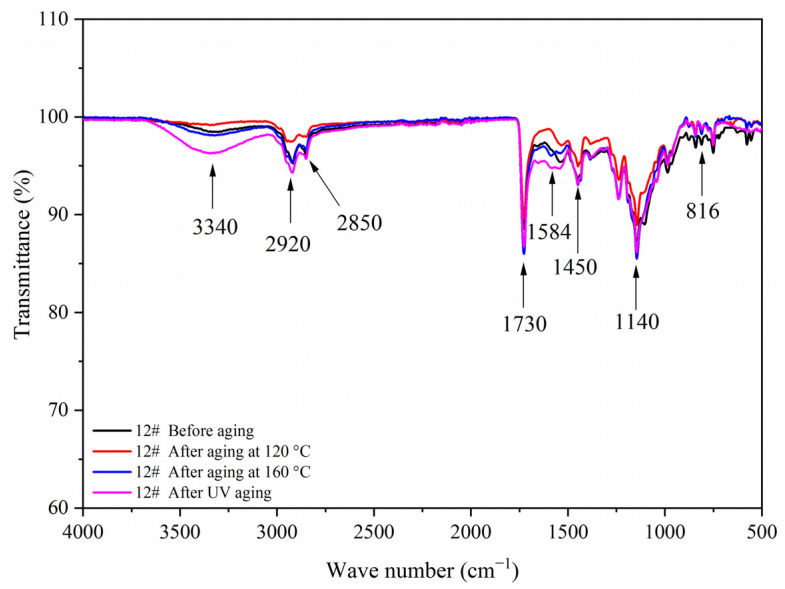
FTIR of sample 12# before and after aging.

Figure 17, Figure 18, Figure 19 and Figure 20 showed the infrared spectra of 5#, 7#, 11#, and 12# before and after aging, respectively. No peak disappeared or appeared before and after aging of the same sample, which indicated that there was no difference in the structure of the coating before and after aging. After the 120 °C aging test, the 5# and 12# coating films with fluorane microcapsules could still achieve thermochromism, but the discoloration effect was lower than that before aging. After the 160 °C and UV aging experiments, the coating films could not achieve thermal discoloration. This is because the molecular chain of fluorane discoloration microcapsules was prone to break under long-term high temperature and ultraviolet light irradiation, so the thermal discoloration function was lost [36].

### 3.6. Self-Repairing Experiment of Coating Film

**Figure 21:**
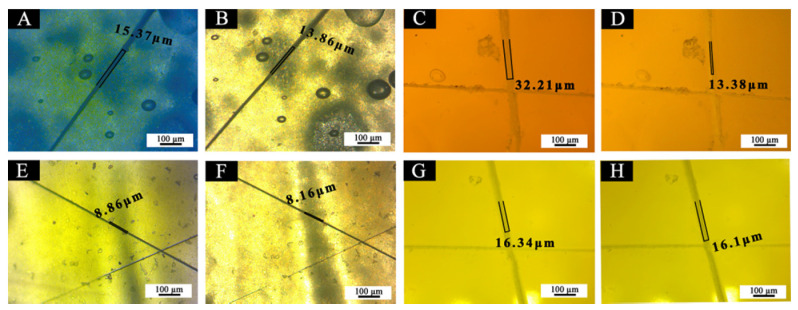
OM of coating films: before self-repairing (**A**) 5#, (**C**) 7#, (**E**) 11#, (**G**) 12#, after self-repairing (**B**) 5#, (**D**) 7#, (**F**) 11#, (**H**) 12#.

The OM images of crack widths of 5#, 7#, 11#, and 12# coating films before and after repairing were shown in Figure 21. Table 5 shows the comparison of scratch gap width before and after self-repairing, according to Figure 21. The crack width of the coating film added with the waterborne acrylic resin microcapsules was reduced after 5 d. The crack width of the best sample (7#) before repairing was 32.2 µm, and the crack width observed again after 5 d at room temperature was 13.3 µm. The gap width of the coating film was reduced by 18.8 µm, and the repair rate was 58.4%. The crack width of 5# coating film before repairing was 15.3 µm, and the crack width observed again after 5 d at room temperature was 13.8 μm, and the gap width was reduced by 1.5 µm. There is no change in crack width before and after repair without adding water-based acrylic resin microcapsules. By contrast, sample 7# of the “primer with 5.0% waterborne acrylic microcapsules and 15.0% fluorane microcapsules” and sample 5# of “primer with 5.0% waterborne acrylic microcapsules and 0% fluorane microcapsules” had a self-repairing effect. When the microcracks occurred in the coating, the wall material of waterborne acrylic resin microcapsules broke, and the core material waterborne acrylic resin was released. With the continuous evaporation of water, isocyanate in polyurethane reacted with hydroxyl groups in waterborne acrylic resin, cross-linking curing occurred at room temperature, carbamate was formed, and homogeneous film with certain mechanical properties was formed, thus, cracks were repaired to a certain extent [37].

Samples 7# and 5# were added with the same content of waterborne acrylic resin microcapsules, and the width of 7# coating film before repairing was much larger than that of 5# coating film, but the crack width after repairing was similar. It was speculated that the two microcapsules were broken in the scratch test, and the amino group in the core material of the fluorane microcapsule reacted with the isocyanate in the waterborne acrylic resin microcapsule, which accelerated the curing of the core waterborne coatings and improved the repair efficiency [38].

## 4. Conclusions

The comprehensive performance of the coating film was better when the 15.0% fluorane microcapsule and 5.0% waterborne acrylic resin microcapsule were added to the primer at the same time. At this time, the coating film changed from yellow to colorless at 32 °C, with a maximum color difference of about 77.9, hardness of 4H, adhesion of grade 0, impact resistance of 12.0 kg∙cm, and elongation at a break of 17.7%. The coating film had good resistance to ethanol, NaCl, and detergent, and the resistance to red ink was grade 2 with slight discontinuous marks, and the self-repairing rate was 58.4%. The coating film with fluorane microcapsules only was unstable during aging, and the color difference changed greatly. Waterborne acrylic resin microcapsules can enhance the aging resistance of the coating film with fluorane microcapsules, and fluorane microcapsules can enhance the self-repairing performance of the coating film with waterborne acrylic resin microcapsules. It was proved that these two kinds of microcapsules can cooperate with each other when they are added into waterborne coatings at the same time. The results provide a technical reference for the development of multifunctional waterborne coating film.

## Figures and Tables

**Table 1:** Orthogonal experiment design of thermochromic self-repairing coating film.

Sample Number (#)	Content of Fluorane Microcapsules (%)	Content of Waterborne Acrylic Resin Microcapsules (%)	Microcapsule Adding Method
1	10.0	5.0	Fluorane microcapsules and waterborne acrylic resin microcapsules were added to the primer
2	10.0	15.0	Fluorane microcapsules and waterborne acrylic resin microcapsules were added to the topcoat
3	20.0	5.0	Fluorane microcapsules and waterborne acrylic resin microcapsules were added to the topcoat
4	20.0	15.0	Fluorane microcapsules and waterborne acrylic resin microcapsules were added to the primer

**Table 2:** Ingredient of thermochromic and self-healing waterborne coatings.

Sample Number (#)	Content of Fluorane Microcapsules (%)	Content of Waterborne Acrylic Resin Microcapsules (%)	Mass of Fluorane Microcapsules (g)	Mass of Waterborne Acrylic Resin Microcapsules (g)	Primer (g)	Topcoat (g)
1	10.0	5.0	0.2	0.1	1.7	2.0
2	10.0	15.0	0.2	0.3	2.0	1.5
3	20.0	5.0	0.4	0.1	2.0	1.5
4	20.0	15.0	0.4	0.3	1.3	2.0
5	0	5.0	0	0.1	1.9	2.0
6	5.0	5.0	0.1	0.1	1.8	2.0
7	15.0	5.0	0.3	0.1	1.6	2.0
8	20.0	5.0	0.4	0.1	1.5	2.0
9	25.0	5.0	0.5	0.1	1.4	2.0
10	30.0	5.0	0.6	0.1	1.3	2.0
11	0	0	0	0	2.0	2.0
12	15.0	0	0.3	0	1.7	2.0

**Table 3:** Range results of the coating film color difference.

Sample (#)	Content of Fluorane Microcapsules (%)	Content of Waterborne Acrylic Resin Microcapsules (%)	Method of Adding Microcapsules	Color Difference
1	10.0	5.0	Fluorane microcapsules and waterborne acrylic resin microcapsules were added to the primer	68.3
2	10.0	15.0	Fluorane microcapsules and waterborne acrylic resin microcapsules were added to the topcoat	55.6
3	20.0	5.0	Fluorane microcapsules and waterborne acrylic resin microcapsules were added to the topcoat	71.0
4	20.0	15.0	Fluorane microcapsules and waterborne acrylic resin microcapsules were added to the primer	81.2
Mean value 1	61.950	69.650	74.750	
Mean value 2	76.100	68.400	63.300	
Range	14.150	1.250	11.450	

**Table 4:** Effect of microcapsule concentration on mechanical properties.

Sample Number (#)	Content of Fluorane Microcapsule (%)	Hardness (H)	Adhesion (Grade)	Impact Resistance (kg∙cm)	Elongation at Break (%)
5	0	2H	0	6	35.0
6	5.0	2H	0	7	37.9
1	10.0	3H	0	8	25.9
7	15.0	4H	0	12	17.7
8	20.0	4H	0	12	9.6
9	25.0	5H	0	13	7.0
10	30.0	5H	0	13	2.2

**Table 5:** Comparison of scratch gap width before and after self-repairing.

Sample (#)	Scratch Width before Repair (µm)	Scratch Width after Repair (µm)	Scratch Width Difference before and after Repair (µm)	Self-Repairing Rate (%)
5	15.3	13.8	1.5	9.8
7	32.2	13.3	18.8	58.4
11	8.8	8.1	0.7	7.9
12	16.3	16.1	0.2	1.4

## Data Availability

Not applicable.
